# A Novel Technique to Identify Intimate Partner Violence in a Hospital Setting

**DOI:** 10.5811/westjem.2022.7.56726

**Published:** 2022-09-12

**Authors:** Azade Tabaie, Amy J. Zeidan, Dabney P. Evans, Randi N. Smith, Rishikesan Kamaleswaran

**Affiliations:** * Emory University School of Medicine, Department of Biomedical Informatics, Atlanta, Georgia; † Emory University School of Medicine, Department of Emergency Medicine, Atlanta, Georgia; ‡ Emory University, Rollins School of Public Health, Hubert Department of Global Health, Atlanta, Georgia; § Emory University, Rollins School of Public Health, Department of Behavioral, Social and Health Educations Sciences, Atlanta, Georgia; ¶ Emory University School of Medicine, Department of Surgery, Atlanta, Georgia; || Georgia Institute of Technology and Emory School of Medicine, Department of Biomedical Engineering, Atlanta, Georgia

## Abstract

**Introduction:**

Intimate partner violence (IPV) is defined as sexual, physical, psychological, or economic violence that occurs between current or former intimate partners. Victims of IPV may seek care for violence-related injuries in healthcare settings, which makes recognition and intervention in these facilities critical. In this study our goal was to develop an algorithm using natural language processing (NLP) to identify cases of IPV within emergency department (ED) settings.

**Methods:**

In this observational cohort study, we extracted unstructured physician and advanced practice provider, nursing, and social worker notes from hospital electronic health records (EHR). The recorded clinical notes and patient narratives were screened for a set of 23 situational terms, derived from the literature on IPV (ie, assault by spouse), along with an additional set of 49 extended situational terms, extracted from known IPV cases (ie, attack by spouse). We compared the effectiveness of the proposed model with detection of IPV-related International Classification of Diseases, 10th Revision, codes.

**Results:**

We included in the analysis a total of 1,064,735 patient encounters (405,303 patients who visited the ED of a Level I trauma center) from January 2012–August 2020. The outcome was identification of an IPV-related encounter. In this study we used information embedded in unstructured EHR data to develop a NLP algorithm that employs clinical notes to identify IPV visits to the ED. Using a set of 23 situational terms along with 49 extended situational terms, the algorithm successfully identified 7,399 IPV-related encounters representing 5,975 patients; the algorithm achieved 99.5% precision in detecting positive cases in our sample of 1,064,735 ED encounters.

**Conclusion:**

Using a set of pre-defined IPV-related terms, we successfully developed a novel natural language processing algorithm capable of identifying intimate partner violence.

## INTRODUCTION

Intimate partner violence (IPV) is defined as sexual, physical, psychological, or economic violence that occurs between current or former intimate partners.^1^ Although men may experience IPV, women are disproportionately affected.^2^ Nearly 30% of women globally have experienced IPV, making it a serious public health concern.^3^ Intimate partner violence is a significant contributor to violence-related injury and a leading cause of femicide, which is the intentional killing of women based solely on their gender.^4^ In the United States one in four women and one in nine men have experienced a severe form of IPV at some point in their lifetime.^5^

Individuals who experience IPV experience both short- and long-term adverse health outcomes such as chronic pain, substance abuse disorder, and mental health disorders.^6^–^9^ People experiencing relationship violence may seek care for IPV-related injuries in healthcare settings, including emergency departments (ED), making recognition and intervention in these facilities critical.^10^–^11^ A recent study revealed that patients experiencing IPV have considerably higher ED visit rates and injury-related hospitalization rates.^12^ Yet IPV is profoundly underdiagnosed in healthcare settings, limiting identification and response efforts. A number of screening tools have been successfully developed to detect IPV in ED settings; however, screening tools are inconsistently used. Emerging efforts have focused on using machine learning to aid in detection of conditions including non-accidental trauma and IPV.^13^–^15^

Information captured in the electronic health record (EHR) including clinical notes, radiology reports, and imaging tests have been widely used to predict adverse outcomes for specific medical conditions. Khurana et al proposed a machine learning algorithm that uses radiologic findings of high-risk injuries (eg, injury location and patterns specific to IPV) to identify patients who are at high risk of IPV.^16^,^17^ Using the 2016 South African Demographic and Health Survey dataset, Amusa et al developed a machine learning model using country-specific, self-reported survey data to capture common characteristics contributing to IPV.^13^ In our study, we propose a novel natural language processing (NLP)-based algorithm using data embedded in the EHR to detect IPV-related ED encounters.

**Population Health Research Capsule**

What do we already know about this issue?

*Intimate partner violence (IPV) is a serious public health concern yet is underdiagnosed in healthcare settings, making identification and intervention difficult*.

What was the research question?

*Could we develop a natural language processing (NLP) algorithm that accurately identifies IPV-related encounters?*

What was the major finding of the study?

*We developed an NLP algorithm that successfully identifies positive cases of IPV with 99.5% precision using unstructured electronic health record data from clinical notes*.

How does this improve population health?

*The NLP algorithm can be used in ED settings in near-real time to identify IPV-related encounters, aid in surveillance mechanisms, and support timely interventions*.

## METHODS

### Study Population

We extracted data from an EHR for all ED encounters between January 2012–August 2020 at a US-based Level 1 trauma center. These structured data included *International Classification of Diseases*, 9^th^ and 10^th^ revisions (ICD-9 or ICD-10) codes, procedure and billing codes, admission diagnosis, disposition, patient status, and date of birth. Unstructured data included chief complaint and all physician and advanced practice provider (APP), nursing, and social worker notes. This research was approved by the Emory University Institutional Review Board (IRB #00432).

### Detecting Intimate Partner Violence Cases

**Figure 1:**
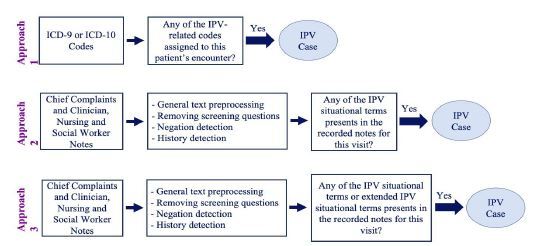
Summary of three methods for developing a natural language processing algorithm to identify intimate partner violence in a hospital setting. International Classification of Diseases, 9th and 10th revisions; *IPV*, intimate partner violence.

To identify IPV-related encounters, we attempted to use structured data, followed by use of the unstructured data. The three iterative approaches used to identify IPV-related encounters are further described in this paper. Figure 1 summarizes the different approaches in this analysis.

### Approach 1: ICD-9/ICD-10 Codes

In the first approach, we identified IPV-related ICD-9 (2012–September 2015) and ICD-10 (October 2015–August 2020) codes (Table 1). In this analysis, if at least one of the ICD-9/ICD-10 codes appeared in an encounter, the encounter was identified as a case of IPV.

### Approach 2: Intimate Partner Violence Situational Terms

Intimate partner violence is socially stigmatized and often undisclosed by those experiencing it; clinicians may also have varying levels of awareness and comfort in dealing with IPV. As a result, ICD-9/ICD-10 codes are inconsistently used and frequently underused. Therefore, we used additional IPV-related situational terms to identify patients experiencing IPV. A total of 23 situational terms were derived from existing IPV literature, including validated terms from IPV risk-assessment instruments and from clinician expertise for use in our second approach (Table 2).^18^–^20^ If any one of the situational terms was captured in a clinical note, the encounter was classified as IPV.

### Approach 3: Intimate Partner Violence Extended Situational Terms

Using a reverse engineering approach, we identified additional IPV-related terms through review of notes from confirmed IPV encounters and derived from the literature. A total of 49 extended terms included specific descriptions of various forms of physical abuse (ie, attack, strike, strangle) (Table 2).^3^,^18^–^20^ If any of the situational or extended situational terms were captured in a clinical note, we classified the encounter as IPV.

### Data Pre-processing: Approaches 2 and 3

A member of the study team completed a manual review of charts identified as positive IPV cases in real time when assessing approaches 2 and 3. During the application of approaches 2 and 3, several text-based scenarios identified in unstructured clinical notes led to false-positive IPV cases. As a result, additional data pre-processing steps were required to prepare the data prior to application of the algorithm. These include general and task-specific text pre-processing steps along with negation and history detection.

### General and Task-Specific Pre-processing

We performed general text pre-processing steps including transforming all text to lowercase and removing numbers, extra white spaces, and words with fewer than two characters. Additionally, prepositions and time indications were removed from the text to make clinical notes consistent. For example, “assaulted last night by her husband” was changed to “assault by husband.” The following text-based scenarios led to false positives: 1) auto-populated IPV screening questions (whether completed or blank); and 2) auto-populated past medical, obstetric, or psychiatric history reflecting a history of IPV unrelated to the identified encounter. As a result, task-specific text pre-processing was required for these scenarios.

### Negation Detection

Encounters in which the patient denied a history of IPV were incorrectly labeled as IPV given the inclusion of IPV terminology. To omit these false positives, we applied a negation detection algorithm, which is a simplified version of NegEx software (SourceForge, San Diego, CA).^21^ In this approach, negation words and terminating tokens are defined. When a negation word was detected, any word between the negation word and the next terminating token was negated. For example, if the text included “Patient denies drug, alcohol use and intimate partner violence,” *denies* was identified as the negation word and *period* was the termination token. Therefore, applying the negation detection algorithm resulted in “Patient denies drug_neg, alcohol_neg use_neg and_neg intimate_neg partner_neg violence_neg.” As a result, such cases were excluded from situational and extended IPV terms and thus not labeled as IPV. Table 3 includes a list of negation words as well as termination tokens in our analysis designed according to the literature.^22^

### History Detection

The algorithm initially detected encounters in which a patient had a history of IPV as described in the text of the EHR (separate from the auto-populated history). Similar to the approach to negation detection, encounters with a history of IPV included in the text were not labeled as IPV as this was not the reason for the ED encounter. For example, “Patient reports a history of IPV during previous pregnancy but not currently” was not labeled as IPV. Punctuation marks were removed at the end of this step. We list IPV history detection tokens in Table 3.

### Natural Language Processing Algorithm Application

To validate the performance of the proposed NLP algorithm for Approach 1 (ICD-9 and IC-10 codes) we cross referenced medical record numbers (MRNs) identified using the predetermined IPV-related ICD-10 codes with the hospital trauma registry for a set time period of 2019–2020. Encounters identified from the trauma registry labeled as positive IPV encounters by ICD-10 codes were manually reviewed by a single reviewer with knowledge of the study’s primary objective and prior training in data abstraction to determine whether the ICD-10 codes correctly labeled IPV encounters. Given the time-intensive nature of manual chart review, we selected this time period (2019–2020) as a pilot to assess the accuracy of this approach, and we used the trauma registry as most patients admitted for an IPV-related injury are admitted to the trauma service. The accuracy of this approach was poor, and thus no further charts were reviewed beyond this time period.

To validate the performance of the proposed NLP algorithm for approaches 2 and 3, manual chart reviews were conducted for the encounters labeled as IPV using situational and extended situational terms. Chart reviews were conducted by a single reviewer with knowledge of the study’s primary objective and prior training in data abstraction. Unlike in approach 1, the trauma registry was not used to narrow review as this would not allow for identification of the specific terminology identified using the NLP algorithm. Rather, manual review was required to identify terminology in the notes of encounters identified as IPV. Manual review was conducted for 25% of the identified IPV cases, and charts were reviewed randomly by year. During the initial manual review process, we determined this approach to be successful at correctly labeling IPV encounters, and thus the percentage of total charts to review (~25%) was determined based on feasibility of manual review (1,798 encounters). Notably, as the reviewer approached this number of charts, the number of false positives was negligible.

## RESULTS

During the study period (January 2012–August 2020) there were 1,064,735 ED encounters (405,303 patients). To identify IPV encounters, we used all ICD-9 and ICD-10 codes and data from structured and unstructured notes to investigate the performance of the three approaches.

### Approach 1: ICD-9/ICD-10 Codes

The first approach using ICD-9 and ICD-10 codes exclusively to identify cases of IPV in a ED setting resulted in the identification of 1,404 IPV encounters representing 1,299 patients over a nine-year time period.

### Approach 2: Intimate Partner Violence Situational Terms

In the next approach, 23 IPV-related situational terms were used to identify IPV encounters. If any of these terms appeared in an encounter’s recorded clinical notes, the encounter was labeled as IPV. This approach yielded 6,437 IPV encounters reflecting 5,280 patients.

### Approach 3: Intimate Partner Violence Extended Situational Terms

Building on the second approach, additional mechanism-related terminology (ie, attack, strike, strangle) was added to the initial 23 terms to identify more IPV-related encounters (defined as IPV extended situational terms). The third approach using IPV extended situational terms identified 7,399 IPV-related encounters representing 5,975 patients. Notably, when comparing approach 1 and approach 3, 96 encounters identified by extended situational terms were also identified by ICD codes (corresponding to 95 patients). The terms that were listed in notes from encounters identified by ICD codes included domestic violence, DV, intimate partner violence, IPV, domestic abuse, domestic violence resources, assault by boyfriend, attack by boyfriend, assault by ex, assault by husband, attack by husband, spouse abuse, domestic dispute, and battered woman.

### Validation of Approaches

For approach 1, the encounters labeled as IPV using ICD-10 codes from 2019–2020 were cross referenced with the trauma registry (552 encounters for 2019 and 2020). Of the ICD-10 codes that labeled positive IPV encounters, 85 MRNs were identified from 2019 and 114 from 2020 from the trauma registry. After completion of manual chart review of the 199 encounters, only 16 of the MRNs identified represented a confirmed encounter for IPV (8%).

For approaches 2 and 3, a random subset of 1,798 (25%) encounters of identified cases were manually reviewed to validate this approach. Nearly all of the 1,798 cases (99.5%) were confirmed IPV encounters; only five (0.3%) reported a history of IPV or domestic violence, two (0.1%) were incorrectly labeled as IPV, and there was a concern of IPV for only one (0.1%) encounter. Relative to the use of ICD codes, both the situational and extended situational terms approaches had significantly improved accuracy in identifying true IPV cases, with extended situational terms identifying more positive IPV cases without a notable difference in identifying false positives.

**Figure 2:**
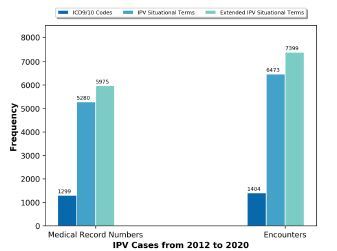
Identified intimate partner violence (IPV) cases using ICD-9/10 codes, IPV situational terms and extended IPV situational terms. *ICD*, International Classification of Diseases, 9th and 10 revisions; *IPV*, intimate partner violence.

The number of IPV cases identified through each approach – ICD-9/10 codes, IPV situational terms, and IPV extended situational terms – are displayed in Figure 2. While an extensive analysis of patient demographic and clinical factors was beyond the scope of this study, we did explore age demographics of patients identified by IPV extended situational terms. Of the 7,399 encounters identified by IPV extended situational terms, most encounters were by adults (ages 22–64; n = 6,378), followed by young adults (ages 14–21, n = 877) and older adults (age >65, n = 144).

## DISCUSSION

This study used EHR data as a means of identifying possible IPV among patients presenting to the ED. Three different NLP approaches were explored to identify IPV in ED settings: 1) ICD-9/ICD-10 codes; 2) a set of 23 IPV-related situational terms; and 3) a set of 49 IPV-related extended situational terms. Among the three approaches incorporated in this study, the use of ICD-9/ICD-10 codes alone identified the fewest IPV encounters over a nine-year time interval (n = 1,404 encounters) with the lowest accuracy. Additionally, based on clinician expertise and anecdotal experiences at the hospital site, this number of cases was significantly lower than expected given the duration of time. Intimate partner violence encounters were significantly undercoded and, in some cases, IPV-related codes were used for non-IPV related encounters (ie, elder abuse). This approach is not sufficient for the accurate and meaningful identification of IPV-related encounters.

The second and third approaches using unstructured EHR data identified a greater number of IPV encounters, generated fewer false positives, and more accurately identified true positive cases. As a result, the third approach using extended situational terms generated the largest number of true IPV encounters, achieving a 99.5% precision. Furthermore, during the manual review of positive IPV cases identified through approach 3, a number of true IPV encounters did not have an associated IPV ICD-9 or ICD-10 code, verifying that these codes are under- or inappropriately used, reifying the need for more expansive detection methods beyond the use of ICD codes alone.

In a study conducted by Chen et al the authors generated an NLP predictive algorithm using radiology reports from confirmed IPV cases.^17^ The IPV labels were identified using IPV injury patterns and predictive words from radiologic findings. The Chen study differed from ours in that it relied only on radiologic findings to develop an algorithm rather than clinical notes. The information obtained in clinical notes provides greater context and IPV-specific terminology and is more inclusive of individuals who may not undergo radiologic imaging. Thus, our algorithm may be able to detect more cases by using a more expansive source of clinical information. Similar to our study, Blosniche et al used clinical notes to identify transgender-related terminology to better identify transgender patients.^23^ The methodology differed in that they first used transgender-based ICD codes to identify patients and then used clinical notes from these encounters to identify transgender-related terms. Th Blosniche study, alongside ours, demonstrates that clinician notes can be an important source of data for labeling encounters that are otherwise difficult to identify or are socially stigmatized. It should also be noted that the purpose of their study was different in that it sought to identify a population (transgender patients) rather than a condition or experience (IPV).

Unstructured EHR data with free-text formatting provides a rich source of information related to the circumstances of medical visits and related health sequelae. The data provided in clinical notes can be an important source of information to identify the social and contextual factors surrounding IPV-related encounters, as well as providing an opportunity to appropriately identify IPV encounters. The main challenge in using this type of data is the unstructured nature of notes, which makes extracting information a complicated task. As a result, application of extensive pre-processing steps was required to ready these data for the screening process. Sequentially building our algorithm grounded first in ICD codes, and then complemented by both situational and extended terms, enabled greater specificity in identifying IPV cases when compared to the use of ICD codes alone; the search and use of relevant terms in clinical notes was key to the success of this approach. Future efforts to improve our algorithm could incorporate active learning to identify a greater number of IPV encounters.^24^ This method is a process of prioritizing the data, which needs to be labeled to improve the overall performance of a predictive model.

Individuals experiencing IPV often seek care in the ED. Therefore, the early and appropriate detection of and response to such cases is critical in disrupting the cycle of abuse including IPV-related morbidity and mortality. The novel NLP-based algorithm we describe here is an innovative tool to use recorded clinical notes and identify victims of IPV in a near real-time setting with accuracy. The algorithm can be used in ED settings to identify victims of IPV for surveillance and intervention purposes. For example, the extent to which coronavirus 2019 (COVID-19) impacted IPV-related health-seeking behaviors in the US is still largely unknown.^25^–^28^ As identification of IPV in health systems is challenging, application of this algorithm could assist with understanding the impact of movement-related restrictions during the COVID-19 pandemic on IPV-related encounters.

When considering potential interventions, documentation of IPV by clinicians may not always translate to the assignment of accurate diagnostic codes, appropriate screening, referral to social work, and/or allocation of immediate and short-term resources and follow-up. The practicality of this novel algorithm is the potential for real-time identification of individuals at risk that could trigger automatic notifications/best practice advisories in the EHR to ensure that appropriate screening, referrals and resources are available to patients. Additionally, this algorithm could be used to develop predictive modeling allowing for the detection of those at risk of IPV. Early detection during hospital encounters could aid in novel injury-prevention strategies, ensuring that those at risk have access to support and social services.

## LIMITATIONS

This study has limitations. All approaches required use of EHRs. While the use of EHRs is now standard in most US hospital settings, one limitation is that any information not captured in the EHR would not be included in our analysis. In our first approach using ICD codes, a number of encounters were found to be unrelated to IPV during manual review, resulting in false positives. Some cases were indicative of elder abuse, reflecting the inaccuracy of relying exclusively on ICD-9 and ICD-10 codes. This limitation inspired the subsequent approaches as these codes are often used inconsistently or inappropriately.

The second and third approaches relied on clinical notes and patient narratives present in the EHR; as a result, the model cannot detect IPV cases if the patient or clinician did not mention or document any of the IPV-related terms included in the algorithm. Similarly, grammatical errors, misspelling, punctuation errors, etc, can impact identification of IPV cases. In future work, deep learning-based natural language models, such as transformers, could be used to overcome these problems and boost the performance and generalizability of the IPV-detection algorithm. To most effectively capture experiences of IPV that were present in the EHR, we applied extensive text pre-processing before searching for IPV situational terms. However, if a patient or clinician stated the history of IPV in a way that was not captured by our history detection algorithm, the proposed NLP algorithm would incorrectly identify that case as IPV.

Third, the set of IPV terms that were incorporated are limited. If a patient uses terminology outside the set of pre-defined IPV situational terms, the algorithm will not identify the encounter. Additionally, some terms may be used in a non-IPV context. For example, *domestic dispute* can be used in IPV encounters but can also refer to a conflict among members of a family (eg, mother and child) and generate false positives. Furthermore, we excluded historical cases of IPV in our labeling to capture only encounters where a patient reported current IPV. As prior IPV is a risk factor for future IPV, excluding these encounters may have missed some potential cases of IPV while at the same time improved specificity of the algorithm for detecting IPV in the current encounter. While the extended situational term approach demonstrated superiority compared to the use of ICD codes alone or the use of situational terms it admittedly still missed some cases.

As conversations about the use of NLP and other technologies continue, debate over what degree of precision or sensitivity is reasonable for a model such as ours is warranted. Further, the 99.5% precision calculation in this study was the result of conducting chart reviews for a random subset of 25% of all identified IPV cases; therefore, this number may change based on the subset of charts manually reviewed. Additionally, our manual chart reviews focused on the number of true-positive and false-positive cases. As we did not review the non-IPV encounters, due to the extremely labor-intensive nature of the task, we cannot comment on the sensitivity or specificity of all the positively and negatively identified IPV cases. From our perspective, missing any cases is unacceptable. In designing any future models researchers should aim to achieve even greater sensitivity to ensure that opportunities to identify and interrupt IPV are not missed.

## CONCLUSION

We developed a natural language processing algorithm that uses an extended list of situational terms for application using unstructured electronic health record data from clinical notes to accurately identify intimate partner violence encounters. This approach was superior to the use of ICD codes or a more limited list of terms. This algorithm has a high precision in detecting cases of IPV and can be incorporated as a decision support system in health system EHRs to identify IPV cases.

## Figures and Tables

**Table 1:** ICD-9 and ICD-10* used to identify cases of intimate partner violence in an emergency department setting.

ICD-9 Codes		ICD-10 Codes	

Code	Diagnosis (Dx) Name	Code	Diagnosis (Dx) Name
995.83	Adult sexual abuse	T76.21XA	Adult sexual abuse, suspected, initial encounter
995.83	Adult rape	T76.51XA	Adult forced sexual exploitation, suspected, initial encounter
995.82	Adult emotional abuse	T76.11XA	Adult physical abuse, suspected, initial encounter
995.81	Adult physical abuse	T74.11XA	Adult physical abuse, confirmed, initial encounter
995.8	Adult abuse	T74.21XA	Adult sexual abuse, confirmed, initial encounter
E967.0	Perpetrator	T74.51XA	Adult forced sexual exploitation, confirmed, initial encounter
E967.9	Perpetrator	T71.9XXA	Asphyxiation due to unspecified cause, initial encounter
994.7	Asphyxiation and strangulation	T71.163A	Asphyxiation due to hanging, assault, initial encounter
-		T71.193A	Asphyxiation due to mechanical threat to breathing due to other causes, assault, initial encounter

* *ICD-9/10*, International Classification of Diseases, 9th and 10th revisions; *IPV*, intimate partner violence.

**Table 2:** Intimate partner violence (IPV) situational terms and IPV extended situational terms to identify positive IPV cases in an emergency department setting.

IPV Situational Terms	IPV Extended Situational Terms
domestic violence, intimate partner violence, spouse abuse, battered woman, domestic abuse, spousal abuse, intimate partner abuse, battered, violence against women, domestic assault, domestic dispute, problems with spouse or partner, maltreatment by spouse or partner, neglect and abandonment by spouse or partner, assault by husband, assault by partner, assault by wife, assault by spouse, assault by boyfriend, assault by girlfriend, assault by significant other, referral to partnership against domestic violence, resources or shelter for domestic violence	intimate partner homicide, femicide, intimate partner death, spousal homicide, ipv, dv, domestic violence resources, assault by so, assault by domestic partner, assault by ex, assault by bf, assault by gf, strangle by boyfriend, strangle by girlfriend, strangle by wife, strangle by husband, strangle by spouse, strangle by domestic partner, strangle by partner, strangle by significant other, strangle by so, strangle by ex, strangle by bf, strangle by gf, strike by boyfriend, strike by girlfriend, strike by wife, strike by husband, strike by spouse, strike by domestic partner, strike by partner, strike by significant other, strike by so, strike by ex, strike by bf, strike by gf, attack by boyfriend, attack by girlfriend, attack by wife, attack by husband, attack by spouse, attack by domestic partner, attack by partner, attack by significant other, attack by so, attack by ex, attack by bf, attack by gf, violence against women

*IPV*, intimate partner violence.

**Table 3:** Negation words, terminations tokens, and history words for a natural language processing algorithm to identify cases of intimate partner violence in an emergency department setting.

Negation words	Termination tokens	History words
"denies", "denied", "deny", "no", "non", "not", "without", "unable"	"?", ".", "−", ";", ":", "+", "and", "but", "complains", "did", "except", "has", "per", "pt", "reports", "secondary", "states"	“history of”, “hx of”, “h/x of”, “ho of”, “h/o of”, “hx”, “h/x”, “h/o”, “ho”
